# Estimating the Stoichiometry of HIV Neutralization

**DOI:** 10.1371/journal.pcbi.1000713

**Published:** 2010-03-19

**Authors:** Carsten Magnus, Roland R. Regoes

**Affiliations:** Integrative Biology, ETH Zurich, Zurich, Switzerland; Utrecht University, Netherlands

## Abstract

HIV-1 virions infect target cells by first establishing contact between envelope glycoprotein trimers on the virion's surface and CD4 receptors on a target cell, recruiting co-receptors, fusing with the cell membrane and finally releasing the genetic material into the target cell. Specific experimental setups allow the study of the number of trimer-receptor-interactions needed for infection, i.e., the *stoichiometry of entry* and also the number of antibodies needed to prevent one trimer from engaging successfully in the entry process, i.e., the *stoichiometry of (trimer) neutralization*. Mathematical models are required to infer the stoichiometric parameters from these experimental data. Recently, we developed mathematical models for the estimations of the stoichiometry of entry [1]. In this article, we show how our models can be extended to investigate the stoichiometry of trimer neutralization. We study how various biological parameters affect the estimate of the stoichiometry of neutralization. We find that the distribution of trimer numbers—which is also an important determinant of the stoichiometry of entry—influences the estimated value of the stoichiometry of neutralization. In contrast, other parameters, which characterize the experimental system, diminish the information we can extract from the data about the stoichiometry of neutralization, and thus reduce our confidence in the estimate. We illustrate the use of our models by re-analyzing previously published data on the neutralization sensitivity [2], which contains measurements of neutralization sensitivity of viruses with different envelope proteins to antibodies with various specificities. Our mathematical framework represents the formal basis for the estimation of the stoichiometry of neutralization. Together with the stoichiometry of entry, the stoichiometry of trimer neutralization will allow one to calculate how many antibodies are required to neutralize a virion or even an entire population of virions.

## Author Summary

A large part of the research on the Human Immunodeficiency Virus focuses on how virus particles attach and enter their target cells, and how entry can be inhibited by antibodies or antiretroviral drugs. Because virus particles are too small to be observed in action the inference of the details of HIV entry has to be indirect—involving the genetic manipulation of virions, and often mathematical modeling. It is known that virus particles establish contact to their target cells with spikes on their surface, and antibodies binding to these spikes can inhibit virus entry. It is not known, however, how many antibodies are needed to neutralize a spike. In this article, we develop a mathematical framework to estimate this number, called the stoichiometry of neutralization, from data obtained in experiments with genetically engineered virions. An estimate of the stoichiometry of neutralization for different antibodies is important, as it will allow us to calculate the amount of antibodies required to abrogate virus replication.

## Introduction

Virions of human immunodeficiency virus (HIV) are coated by a lipid bilayer. Trimers of the dimeric envelope proteins (*Envs*) gp120 and gp41 are inserted into this membrane [3]–[5]. These trimers, often also referred to with the more general term *spikes*, can bind to CD4 receptors [6],[7]. After successful engagement of CD4, the envelope trimer undergoes conformational changes that allow a coreceptor, most commonly chemokine receptors CCR5 and CXCR4, to bind [8]. Binding to the coreceptor initiates a series of rearrangements in the viral envelope protein gp41, which upon insertion of the fusion peptide into the cellular membrane brings together viral and cellular membrane and triggers the fusion process. Possible targets for neutralizing antibodies have been identified over the past decades and are restricted to accessible conserved regions on the Env trimers [9],[10].

Estimating the number of monoclonal antibodies or inhibitory molecules needed to block a single trimer together with estimates of other parameters that characterize the molecular interaction of the virus with its target cells and antibodies, may eventually allow us to predict the antibody concentrations required to inhibit viral replication *in vitro* and within the infected individual. This quantitative understanding of neutralizing antibody activity will aid the development of antibody vaccines and entry inhibitors.

In this paper, we develop a mathematical framework to estimate how many antibodies are needed to neutralize a single trimer. This number is referred to as *stoichiometry of trimer neutralization* or short *stoichiometry of neutralization*. The mathematical models, which we introduce here, are based on the models we developed for the analysis of the number of trimers required for cell entry [1]. As for the stoichiometry of entry, we investigate models differing with respect to the biological assumptions about the exact molecular mechanisms involved in the generation of pseudotyped virions. To illustrate how to use our model to estimate the stoichiometry of neutralization, we reanalyze previously published data by [2].

## Models

### Experimental setup for the determination of the stoichiometry of neutralization

Here, we briefly introduce the experimental setup for the determination of the stoichiometry of neutralization, in particular those aspects relevant for the development of the mathematical models in the next section. The experimental setup is described in more detail in [1],[2],[11].

Envelope-pseudotyped HIV virions are generated by transfection of virus producer cells (293T) with a set of plasmids. One plasmid provides all the genetic information to assemble infectious virions with the exception of the viral envelope. The genetic information for viral envelope proteins is provided on separate plasmids. A third plasmid encodes for the firefly luciferase reporter gene under the control of HIV LTR, which allows rapid detection of infected target cells. The resulting virions contain viral envelope proteins and are infectious but are only capable of completing one infection cycle, as the genetic information packaged into the virions lacks essential genes.

To study the stoichiometry of neutralization pseudotyped virions with mixed envelope proteins are generated. Hereby, plasmids encoding for wild-type envelope proteins are transfected along with plasmids encoding for neutralization-resistant envelope proteins. As a result, the plasmid pool in the producer cell consists of a mixture of wild-type and mutant envelope proteins. Proteins from this pool trimerize and, as a consequence, a fraction of the envelope trimers are wild-type/mutant hetero-trimers. We denote the fraction of mutant envelope protein encoding plasmids by 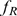. The mutant envelopes harbor only one (or few) amino acid changes compared to the wild-type that render them resistant to a specific neutralizing antibody. Otherwise the mutant envelope proteins are fully functional and can form functional hetero-trimers [12].

The infectivity of these pseudotyped virions with mixed envelope protein trimers is then measured. Before these virions infect target cells, they are saturated with monoclonal antibodies that bind to all wild-type envelope proteins. As a consequence, only mutant envelope proteins can take part in attachment to CD4-receptors. In this assay, infectivity is measured via the luciferase reporter gene, which is expressed upon infection of susceptible target cells. The luciferase activity (measured as emitted relative light units) in the infected cell population is proportional to the number of virions that successfully entered and integrated into a cell. The infectivity is normalized to a virus stock that contains 100% wild-type Envs. Similar to the study of the stoichiometry of entry [1], the relative infectivity, RI, is determined for different fractions 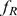 of mutated envelope encoding plasmids.

### Mathematical models for trimer neutralization

The mathematical models to infer the stoichiometry of (trimer) neutralization, 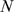, incorporate the combinatorial aspects of the assembly of pseudotyped virions with mixed envelope proteins and the infection of cells in the infectivity assay.

One of the most important input parameter in all of these models is the distribution of the number of trimers on the surface of virions. We include this distribution in a generic form with the parameters 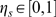, 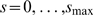, where 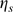 denotes the fraction of virions with 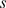 trimers. Note that this distribution only describes the numerical and not the spatial distribution of trimers on the virion's surface.

For the *basic model* we assume that the envelope proteins to be assembled into trimers are sampled out of an envelope pool. The fraction of mutated envelope proteins in this pool is equal to the fraction of mutant Env encoding plasmids in the transfection medium, 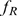. Trimers are formed perfectly randomly from the envelope proteins in the pool, i.e. the number of mutated Env proteins is binomial distributed. Virions can infect a cell if they have at least 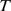 functional trimers.

In the four model extensions we relax different assumptions of the basic model.

- In the *imperfect transfection model* we allow the fraction of mutant envelope proteins in the envelope pool to differ from the fraction of mutant Env-encoding plasmids.
- For the *segregation model* we relax the assumption of binomial-distributed trimer assembly, i.e. the formation of trimers with only wild-type or mutant envelope proteins becomes more likely.
- In the *proximity model*, we assume that trimers have to be sufficiently close to each other to engage with the CD4 receptor on the target cell.
- In the *soft threshold models* we relax the assumption of a strict thresholds. Since our models involve two threshold parameters, the stoichiometry of entry and the stoichiometry of neutralization, we can formulate two types of soft threshold models.

**Figure 1:**
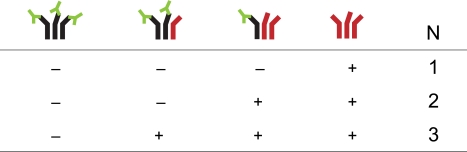
Dependence of the stoichiometry of neutralization, 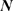, on the trimer's infectiousness. Wild-type envelope proteins are colored black, mutant envelope proteins red and antibodies green. Due to saturation with antibodies prior to the infectivity experiments, all wild-type envelope proteins are assumed to be bound. Functional trimers are marked with “+”, non-functional ones with “−”.

Which virions end up infecting a cell? To answer this question we first have to zoom in on the trimeric level. A trimer is called *functional* if it is able to take part in mediating cell entry. As virions are saturated with antibodies before the infection experiments, this ability is dependent on the stoichiometry parameter 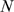. In the absence of antibodies, both mutant and wild-type Envs are assumed to be perfectly functional and give rise to infectious particles. In the investigated setup however, antibodies bind to wild-type Envs and all wild-type Envs are assumed to be bound by one antibody. If a trimer has 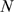 or more wild-type envelope proteins, this trimer is neutralized. Hence, in this setup only trimers with more than 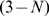 mutated envelope proteins are functional trimers. Figure 1 gives an overview of functional and non-functional trimers depending on the stoichiometry of neutralization 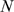. Here lies the important difference between the scenario studied in our work on HIV-entry [1] and the assays to estimate the neutralization parameter [2]. For estimating the entry parameter a mutation was used which renders the complete trimer binding-incapable, i.e. only trimers without any mutated Env protein are functional ones. In the neutralization assay, both wild-type and mutant Envs are infectious and only wild-type Envs can be rendered non-infectious by binding neutralizing antibody.

Not all virions that can potentially infect a cell end up in successfully infecting a cell. We call a virion *infectious* if it has the potential to infect a cell. Therefore it has to fulfill special conditions concerning the number of functional trimers which depend on the model and which are defined for every model separately. We assume that every infectious virion has the same probability to infect a cell independent of the number of functional trimers. Since we study the infectivities of a mixed virion stock in comparison to a wild-type stock this quantity cancels out in the calculations.

#### Basic model for the neutralization assay

Let 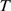 be the stoichiometry parameter of entry as described in [1], i.e. the number of trimers needed for attachment to target cell receptors, fusion and release of the virus' genetic material into the target cell. Let 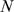 be the stoichiometry parameter of neutralization, i.e. the minimal number of antibodies needed to render a trimer non-functional. Since monoclonal antibodies are used, each antibody can only bind to a specific region of the envelope protein and 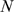 equals either 1,2 or 3.

Let us assume that each envelope protein has the same chance to be selected out of the envelope pool during trimer assembly. Only trimers with more than 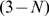 mutated envelope proteins are functional (in this case, the trimer has less than 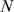 wild-type Envs). Hence, the probability that a trimer is functional, 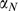, is:
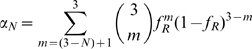
(1)Each trimer is assembled independently and for a virion with 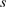 trimers on its surface, the probability that it has 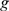 functional trimers is:
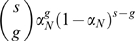
(2)In the basic model the condition for an infectious virion is the following: A virion is infectious if there are at least 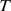 functional trimers (trimers with more than 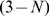 mutated envelope proteins) on its surface. The probability that a virion with exactly 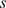 trimers is infectious is equivalent to the probability that it has at least 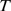 functional trimers. This can be calculated as:
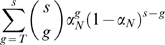
(3)The relative infectivity obtained by experiments is a comparison of the emitted light of an infectivity assay with a pseudotyped virus stock and the emitted light of an infectivity assay with a wild-type virus stock. Hence, the relative infectivity is a function of the fraction 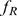 of mutated Env encoding plasmids. Wild-type virions have only functional trimers, and therefore all virions with more than 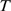 trimers are infectious. Since the probability that a virion has 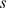 trimers is 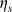, the fraction of infectious wild-type virions is 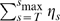. The probability that a virion with 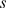 trimers is infectious in equation 1 has to be weighted by the probability 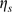 that a virion has 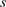 trimers and we obtain the following analytical expression for the relative infectivity:
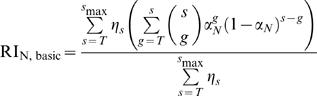
(4)


Using equation 4, we can infer the stoichiometry parameter of neutralization 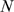 from the observed relative infectivity for various values of 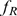, knowing the distribution of trimer numbers 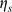 and the stoichiometry of entry. It is also possible to estimate the two stoichiometry parameters 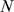 and 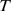 simultaneously.

Yang et al. studied the neutralization sensitivity 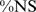 [2]. This is the percentage of virions that can be neutralized or in other words the percentage of virions which can not infect. Hence, the relative infectivity is
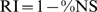
(5)


#### Imperfect transfection

As in the basic model, a virion is infectious if it has at least 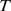 functional trimers. Each trimer has to have more than 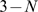 mutated envelope proteins to be functional. In contrast to the basic model, we do not equal the fraction of mutated Env encoding plasmids with the fraction of mutated envelope proteins in the Env pool. Two mechanisms can result in a difference between these two fractions: a) only small or variable numbers of plasmids could enter the transfectable cell or b) different quality of plasmid preparation can lead to differential expression of the Env proteins.

The variation of the fraction of envelope proteins in the Env pool is modeled as a 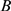-distributed random variable with mean 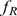. As in the entry model (cf. [1]), the variance 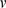 of this distribution is defined as
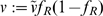
(6)with a parameter 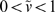, called *variance coefficient*. We still assume binomial trimer assembly. Since the fraction of mutant envelope protein is now 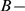
distributed, the probability that a functional trimer is formed in the imperfect transfection model is an integral. The integrand is the same as equation 1, weighted with the probability density function of the 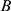-distribution with mean 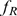 and variance 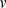, denoted by 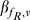:
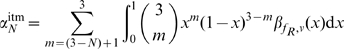
(7)The relative infectivity can now be computed by replacing the probability of forming a functional trimer in the basic model, 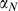, with the probability to form a functional trimer in the imperfect model, 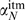:
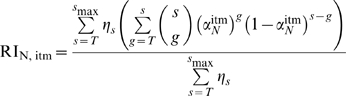
(8)This model can be fitted to the data of Yang et al. [2], by either using the estimated parameters for 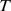 and 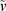 or by estimating 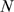, 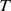 and 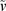 simultaneously.

#### A model allowing for segregation of envelope proteins within transfected cells

As in the basic and the imperfect transfection model, a virion is infectious if it has at least 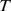 functional trimers on its surface. A trimer is functional, if there are more than 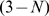 mutated envelope proteins.

In the basic model, the formation of trimers is assumed to follow a binomial distribution. This means that to form a trimer the envelope proteins are sampled from the envelope pool within the cell and the probability to chose one trimer is equally likely for every trimer in this pool. There is evidence that, when two different HIV-1 glycoproteins are expressed in the same cell, hetero-trimers are formed and recruited to the surface of virions [13],[14]. This phenomenon was even observed for coexpressed HIV-1 and HIV-2 envelope proteins [12] and for mixed HA-trimers in influenza A strains [15]. Despite empirical evidence for the existence of hetero-trimers, it is to date unclear if their frequency is consistent with a process of trimer formation that follows a binomial distribution (i.e., loosely speaking, is “perfectly random”). Therefore, in the segregation model, we assume the process of trimer formation to be skewed towards the formation of homo-trimers.

We include the *segregation parameter* 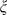, 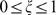. 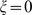 correspond to no segregation (and this is equivalent to the basic model) and 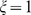 stands for full segregation, i.e. no hetero-trimers are formed.

The probability to draw a mutated envelope protein out of the Env pool is 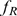, the probability to get a wild-type protein in the first draw is 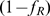. The probability that the second Env is a mutant given the first Env is a mutant is assumed to be 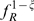. Since 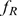 is less than 1, 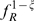 is greater than 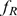 (except for 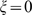, in this case it is equal). The probability that the second Env is a wild-type given the first one is a mutant Env is 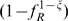. These probabilities are also valid for the third envelope protein. Similar to this definition, the probabilities that the second (third) envelope protein is a wild-type given the first one is a wild-type envelope equals 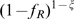, respectively the probability that the second (third) Env is a mutant given the first one is a wild-type is then 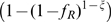. Now we can derive the probabilities that a trimer has 1,2 or 3 mutant envelope proteins, called 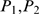 respectively 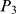:
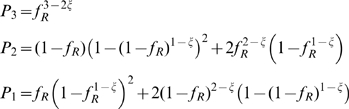
(9)


If 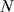 equals 1, only trimers with 3 mutant Envs are infectious. If 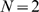, trimers with 2 or 3 mutant Envs and if 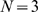, trimers with 1, 2 or 3 mutant Envs are infectious. The probability that a trimer is infectious is therefore
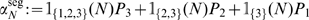
(10)with 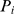 defined in equation 9 and 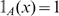 for 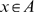 and 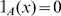 else.

Let 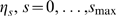 be the probability that a virion has 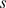 trimers. Hence, the relative infectivity is:
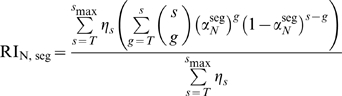
(11)


For fitting this model to the data of Yang et al. [2] one can either use the parameters we estimated in [1] for 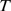 and 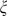 or estimate them beside the stoichiometry of neutralization.

#### Correcting for proximity requirements

In the proximity corrected model, the definition of a functional trimer given in the basic model is valid once again, i.e. there have to be more than 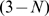 mutated Env proteins in the trimer. This happens with the probability 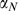 described in equation 1, i.e. we drop the segregation assumption.

In the basic model, a virion is infectious if there are at least 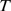 functional trimers independent of the trimers' location on the virion's surface. This assumption is reasonable if trimers can move freely on the cell surface or if trimers are recruited to the site of contact between the virion and the target cell. Some studies using cryoelectron microscopy tomography suggest indirectly that trimers can indeed move freely [16],[17]. On the other hand, Zhu et al. [18] conclude from their study, that trimers seem to have fixed positions on the surface. To model a scenario with fixed trimer positions, we assume, that there has to be a group of at least 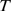 trimers that are sufficiently close together. This group of trimers then establishes the contact between the virion and the target cell. The critical distance, i.e. the maximal distance between each pair of trimers within this group, is denoted with 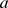.

Deriving an analyzable mathematical expression for the relative infectivity is impossible, so we simulate 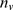 virtual virions with randomly distributed trimer numbers where the probability that a virion has 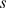 trimers equals 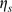. The probability to form a functional trimer is 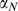, defined in equation 1. The number of functional trimers on a virion with 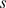 trimers is therefore binomial distributed with the parameters 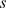 and 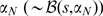. The functional trimers are now distributed randomly on the surface of a spherical virion. If there are at least 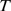 functional trimers with a pairwise distance less than the distance parameter 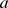, the virion is counted as an infectious one. To obtain the relative infectivity for a certain fraction 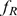 of mutated Env encoding plasmids, we simulate the number of infectious virions with the value of 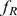 and compare that to the number of infectious virions simulated with 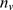 not neutralized wild-type virions. For a more detailed description of the simulation procedure confer the explanation of the simulation in the proximity corrected model for HIV-entry in [1].

#### Soft threshold models

In the previous models we have assumed, that a virion is either infectious or not. Virions with at least 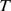 functional trimers are infectious in the basic and segregation model, and in the proximity corrected model these 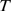 functional trimers have to be sufficiently close to each other. However, one can imagine that all virions are able to infect target cells but not with the same probability, e.g., a virion with 10 functional trimers infects a target cell with two times the probability that a virion with 5 functional trimers end up infecting a cell.

To integrate this idea into our model, we assume that the probability of infectiousness follows a Hill-function. This means that the probability that a virion with 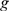 functional trimers is infectious equals
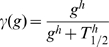
(12)


This definition allows the analysis of a very broad spectrum of infectiousness models. The parameter 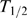 is the number of functional trimers for which the infectiousness probability is 0.5. We assume this parameter to be continuous and bounded by 100, because the maximal number of trimers on a virion is assumed to be 100. The parameter 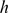 determines the steepness of the infectiousness curve (cf. [1]).

There are two possibilities to define a functional trimer. The first is the definition we used in the basic and proximity corrected model, namely that a trimer is functional, when it has less than 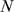 wild-type envelope proteins (i.e. more than 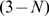 mutant Env). The model is described in equation 13. The second model includes a soft threshold on the trimeric level and is described by equations 14 and 15.

As in the previous models the number of trimers on the virion's surface is distributed according to 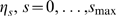.

**Figure 2:**
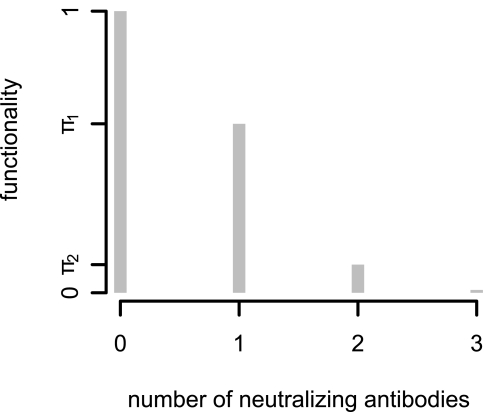
Functionality, i.e. probability of a trimer neutralized by some antibodies to take part in attachment to virus.

- A soft threshold for entry: A trimer is functional, if there are more than 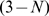 mutant envelope proteins. A trimer with 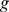 functional spikes is infectious with the probability 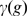, defined in equation 12. Since all virions with at least 1 trimer can be infectious, for the relative infectivity one has to sum over all possible trimer numbers and the possible numbers of functional trimers. Therefore, the relative infectivity is:
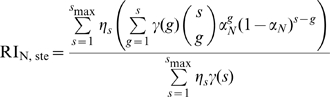
(13)
- A soft threshold for entry and neutralization: Here we relax the assumption that a trimer needs a certain number of antibodies to be neutralized. It is now assumed that the functionality of a trimer, that is the ability to bind to a target cell receptor, is only reduced by antibody-binding. A trimer to which no antibody is bound has a functionality of 1, and a trimer bounded by 3 antibodies is completely neutralized, i.e. its functionality is 0. The functionality of trimers with one respectively two antibodies are 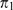 resp. 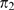, with 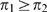. This concept is illustrated in figure 2. Antibodies can only bind to wild-type envelope proteins. This means that the number of antibodies bound to a trimer equals the number of wild-type Envs. The mean functionality can now be calculated by weighting the functionality parameters by the probability of having 0,1,2 resp. 3 wild-type envelope proteins. Hence, the mean functionality 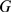 is:
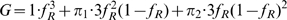
(14)

The expected functionality of one trimer has to be multiplied by the number 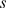 of trimers on a virion to obtain the expected functionality of a virion with 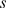 trimers. The infectiousness can now be calculated with equation 12 and 14 as:
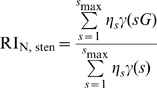
(15)


Equations 13 and 15 are used to fit the soft threshold models to the data of Yang et al. [2]. For the soft threshold model for entry, we can use the parameters obtained in [1], but we also can estimate 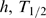 and 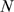 simultaneously. The soft threshold model for entry and neutralization is only fitted under the assumptions, that the parameters for 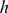 and 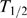 estimated in [1] hold true, i.e. only 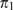, 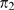 and 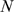 are estimated.

The definitions of all model parameters are summarized in table 1. The expressions in equations 4, 8, 11, 13 and 15 were implemented as functions in the R language for statistical computing [19]. The proximity corrected model is only simulated, due to the lack of an analytical expression for this model. To reduce runtime, the algorithm was implemented in C and loaded into R as a shared library. The programs can be obtained upon request.

**Table 1:** Parameter definitions.

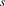	number of trimers on virion
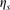	probability that virion has 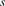 trimers
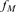	fraction of dominant negative Env-mutant encoding plasmids
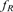	fraction of antibody-resistant Env-mutant encoding plasmids
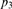	probability of forming a “functional” trimer in the entry assay, 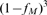
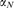	probability of forming a “functional” spike in the neutralization assay, see equation (1)
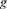	number of “functional” trimer on virion
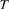	number of trimer-receptor interactions needed for entry
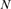	number of antibodies-Env interactions required to neutralize one spike
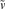	variance coefficient (imperfect transfection model)
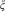	segregation coefficient ranging from 0 (no segregation) to 1 (full segregation)
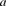	maximal distance of trimers required for cooperation in an infective cluster
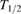	Number of trimers for which infectivity is 1/2
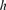	softness parameter (Hill coeficient) in the soft threshold model

## Results

### Effect of the stoichiometry of neutralization 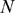 and the distribution of trimer numbers 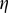 on the relative infectivity

In this section, we first analyze the effects of the input parameters on the predicted relative infectivity. We show predictions for the basic model in detail. Reversing these predictions, we estimate the stoichiometry of neutralization 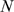 below by fitting our mathematical models to data obtained by experiments of Yang et al. [2].

The relative infectivity RI increases with the fraction of neutralization resistant envelope proteins 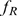. This is quite intuitive because, the more neutralization resistant envelope proteins exist in the transfectable cell, the more trimers with a high number of mutant envelopes are assembled. These trimers are more likely to be functional in the presence of antibody.

**Figure 3:**
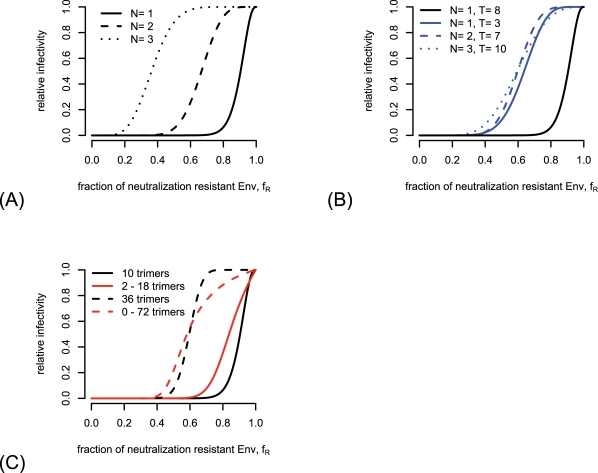
The relative infectivity in the basic model predicted by equation 4 as a function of the fraction 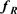 of neutralization resistant envelope proteins. For plot (A) and (B) we assume that each virion has exactly 10 trimers. For plot (A) and (C) the stoichiometry parameter of entry 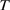 equals 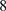, according to our estimates in [1]. (A) Dependence of the relative infectivity on the stoichiometry parameter 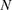. (B) Dependence of the relative infectivity on the stoichiometry of entry 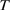. (C) Dependence of the relative infectivity on the mean and variance of trimer numbers. For this plot the stoichiometry parameter 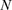 is set to 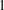. Solid lines are based on a mean number of trimers equal to 10. Dashed lines have a mean trimer number of 36. For the black curves the number of trimers is exactly 10 respectively 36 and the distribution of trimer numbers for the red curves are discrete uniform distributions with 2 to 18 respectively 0 to 72 trimers.

The higher the stoichiometry of neutralization 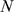, the more the relative infectivity curve is shifted to the left (see figure 3 (A)). For example, let us assume only virions with exactly 10 trimers of which 8 trimers are needed for entry (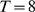). If three antibodies are needed to neutralize one trimer 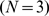 a trimer with one or more mutant envelope proteins is functional. For a fraction of neutralization resistant envelope proteins 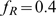, the probability for a functional trimer is 78.4% and this leads to a relative infectivity 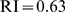 (see figure 3 (A)). In contrast, the relative infectivity for 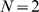 and 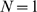 are almost 0 due to the small probabilities for functional trimers. Only 35.2% of all trimers are functional if 2 antibodies are needed for neutralization (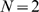), and 6.4% are functional for 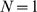.

The stoichiometry of entry 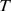 describes the minimal number of trimer-target cell receptor interactions needed to mediate cell entry. If only few trimers are necessary for attachment and entry, the probability that a virion has enough functional trimers is much higher than for a large number of trimer–target interactions 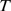. Therefore the predicted relative infectivity decreases with the stoichiometry of entry 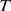 (cf. the blue and black solid curves in figure 3 (B)).

A problem for the estimation of the stoichiometric parameters is that there are various combinations of 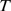 and 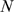 which give rise to very similar predicted relative infectivities. As an example, let us assume a virion population with exactly 10 trimers per virion. For this situation the stoichiometry parameter pairs 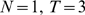; 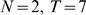 and 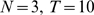 predict similar relative infectivity values (see the blue curves in figure 3 (B)). This suggests that it is not advisable to estimate both stoichiometry parameters 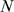 and 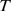 simultaneously (see also below).

As shown in figure 3 (C) mean and variance of the distribution of trimer numbers 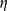 play also important roles for the predictions of the relative infectivity. The higher the mean trimer number, the faster the relative infectivity increases. The variance of the trimer number distribution changes the smoothness of the predicted relative infectivity curve. Since parameter estimations are based on the predicted relative infectivites, it is necessary to include as much information as possible about the distribution of trimer numbers. [18] investigated trimers on HIV-1 virions and found trimer numbers of 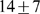 trimers on a virion's surface. Since we do not have more detailed information about trimer number distribution, our estimates are based on a discretized 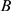-distribution with mean 14 and standard deviation 7 (see figure 5 in [1]).

### Effects of the variance and segregation coefficient

Essential for the estimation of the stoichiometric parameters is the presence of hetero-trimers. As one can see in figure 1 wild-type homo-trimers are neutralized for every stoichiometry of neutralization 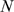 and mutant homo-trimers are not neutralized for any 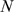. Therefore, most of the information about the stoichiometry of neutralization is contained in the infectivity of virions with hetero-trimers.

**Figure 4:**
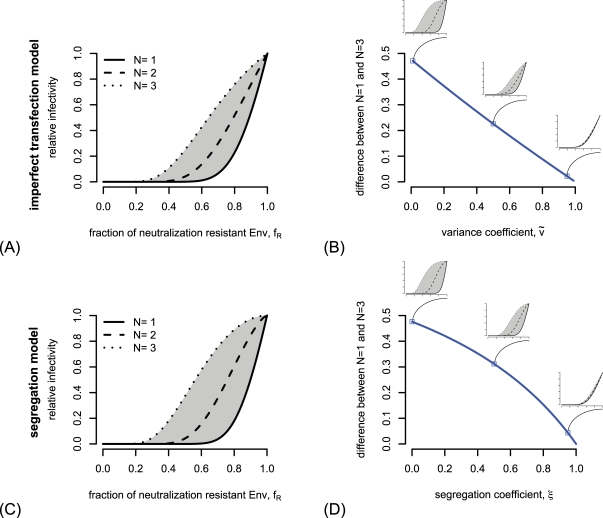
Loss of distinguishability of estimates of the stoichiometry of neutralization 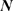
in the imperfect transfection model and the segregation model. (A) Dependence of the predicted relative infectivity in the imperfect transfection model on the stoichiometry parameter 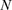, the entry parameters for this figure are 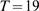 and 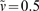. (B) The area between predictions for 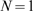 and 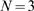 is depicted in dependence of the variance coefficient 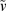. The decrease of this area size with increasing 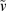 makes the differentiation between different stoichiometries of neutralization difficult for high values of 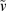. (C) and (D) show the same phenomenon for the segregation model. The parameters for (C) are 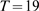 and 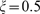. The stoichiometry of entry for (B) and (D) is 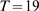.

In the imperfect transfection model, we introduce the variance coefficient 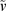 to allow the envelope pool to vary from the fraction of antibody-resistant Env-mutant encoding plasmids 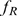. A high 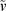 corresponds to a scenario in which almost all cells are transfected exclusively with either wild-type or mutant Env encoding plasmids. As the presence of hetero-trimers is crucial for the determination of the stoichiometry of neutralization, the distinguishability between different estimates of the stoichiometry of neutralization decreases with increasing 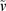. This effect is depicted in figure 4 (A) and (B). From this figure it becomes clear that for variance coefficients 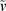 close to one different values of the stoichiometry of neutralization lead to almost identical predictions of the relative infectivity and make a reliable estimate for the stoichiometry of neutralization impossible.

In the segregation model, the segregation coefficient 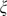 allows a deviance from the binomial sampling from envelope proteins out of the envelope pool. When 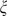 is very close to 1, almost only homo-trimers are formed. Therefore the distinguishability between the predictions of the relative infectivity for the different stoichiometries of neutralization 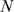 decreases with increasing 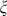 (see figure 4 (C) and (D)). As for high values of 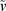, the estimation of the stoichiometry of neutralization is extremely difficult when the segregation parameter is close to 1.

### An example of parameter estimation

To estimate the stoichiometry of neutralization and the other model parameters, we re-analyze data obtained in [2]. Yang et al. investigated 4 different HIV1-strains (ADA, YU2, HXBc2 and KB9) which in sum had 11 different envelope glycoprotein mutants that rendered them insensitive to one or several of the 9 different neutralizing antibodies (b12, 2F5, 2G12, 1121 F105, F91,15e,17b and 48d). Infectivity and neutralization was studied on two different target cell types (Cf2Th-CD4/CCR5 and Cf2Th-CD4/CXCR4). In total, 15 different virus antibody combinations were available for our reanalysis [2]. To demonstrate how our models can be used to derive the stoichiometry of neutralization, we treat this data set in two different ways. First, we include all data points into our estimation. This assumes that all antibodies have the same stoichiometry. However, it could be possible, that the stoichiometry of trimer neutralization varies between different antibodies, i.e. for one type of antibodies only one antibody is sufficient for trimer neutralization whereas for another sort of antibodies two or three abs could be needed to neutralize one trimer. For this analysis, a statistically sufficient number of experiments for the same combination of viral strain, envelopes mutation, antibody and target cell would be required. Since the data set [2] is not sufficient to analyze single antibody-virus combinations, we divide these combinations into 5 groups according to the antibody binding sites. Antibodies F105, b12, 15e and F91 interfere with the CD4 binding site [20]–[23] and are classified as *CD4BS-group*. Antibodies 17b and 48d bind to a highly conserved region induced upon CD4 engagement which is important for gp120-chemokine receptor interaction [24],[25] and belong to the *CD4i-group*. The other three monoclonal antibodies have different binding sites and could not be grouped together. These are *2F5* that binds a linear gp41 epitope proximal to the viral membrane [26], *2G12* that recognizes a carbohydrate-dependent epitope on the gp120 surface [27] and antibody *1121* that recognizes the gp120 V3 loop (ImmunoDiagnostics, Inc.).

We first assume that model parameters for entry derived in [1] are valid for the neutralization assays data from Yang et al. [2]. Under this assumption, we analyze the data either pooling over all antibody-virus combinations, or stratifying with respect to antibody binding site (grouped data). Then we also estimate the parameter of neutralization along with the parameters of entry for the different models, and compare these estimates with the estimates for the neutralization parameter alone.

#### Estimation of the stoichiometry of neutralization

First, we estimate an average stoichiometry parameter 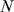 by pooling all data over different antibody-virus combinations. Thus, in our models, we assume that the number of antibodies, which neutralize one trimer, is the same for all antibodies.

**Figure 5:**
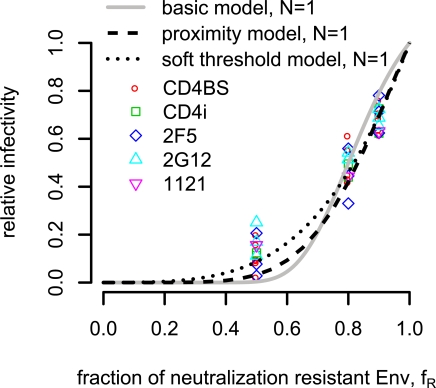
Relative infectivity curves for the best estimate of the stoichiometry of neutralization 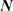 in the different models. The imperfect transfection model and the segregation model are omitted due to the lack of reliability of the estimates. In the other models, the best fit is obtained for 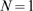. The entry parameters are included from the estimation of the entry parameters in [1]: 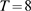 for the basic model, 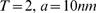 for the proximity model and 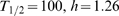 for the soft threshold model.

Assuming that the stoichiometry parameter of entry 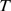 equals 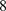, we estimate 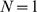 for the **basic model** (see figure 5) with more than 99.9% confidence, determined in a bootstrap procedure with 1000 replicates.

In [1], the estimated parameters for the **imperfect transfection model** are 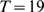 and 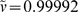. Including these values in fitting the imperfect transfection model to Yang's neutralization data, we obtain the best fit with 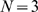. This model extension describes the data significantly better than the basic model (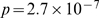). However, the estimation is not very reliable. In a bootstrap procedure only 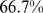 of all the bootstrap estimates are 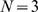, whereas 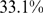 are 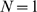 and 0.2% are 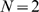. The reason for the high uncertainty in the estimate is the high variance coefficient 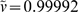 (see also the explanations in the subsection “Effects of the variance and segregation coefficient”).

There are similar problems with the estimation of the stoichiometry of neutralization 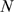 in the **segregation model**. The parameters estimated for entry in [1] are 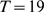 and 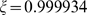. This value is very close to 1 and therefore the estimation of 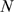 is not reliable. This is also reflected in the uncertainty of the estimates: Although the best estimate is 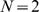 and explains a higher fraction of the variability in the neutralization data by Yang et al. [2] (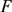-test, 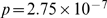), a bootstrap procedure results in 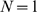 in 21.3%, 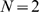 in 41.5%, and 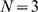 in 37.2% of all replicates (see also the explanations in the subsection “Effects of the variance and segregation coefficient”).

For the entry and the distance parameter in the **proximity model**, the best fit is obtained for 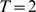 and 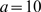
nm (if we assume the diameter of HIV-1 virions to be 100nm). The stoichiometry of neutralization, for which the predictions for the relative infectivity fits the data the best, is 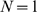 (see figure 5) with 100% confidence (bootstrap procedure). The proximity model fits the data significantly better than the basic model (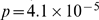).

The **soft threshold model for entry** has two parameters which are important for the predictions of the relative infectivity in the entry process: 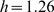 and 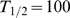. Assuming these parameters, the stoichiometry parameter for neutralization is 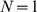 (see figure 5) and a bootstrap routine gives 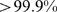 confidence in this estimate. This model extension is significantly better than the basic model 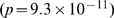. When fitting the **soft threshold model for entry and neutralization** to the data, the functionality parameters 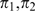 for one respectively two antibodies are both 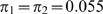. This means that the trimer's ability to mediate cell entry almost vanishes when one antibody binds to the trimer. The improvement of the predictions for the relative infectivity by including a soft threshold for the functionality of trimers is not significant (p = 0.08). We therefore disregard this model from now on.

We also tested, if different antibody groups have different stoichiometries of neutralization. To this end, we stratified the data set with regard to 5 groups corresponding to different binding sites. This model extension did not fit the data significantly better than the model assuming 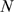 to be equal across binding groups. Furthermore, all estimates for 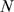 were 1 in the models that allow for reliable estimation (the basic, proximity, and soft threshold models).

#### Simultaneous estimation of the stoichiometry of entry and neutralization

It is also possible to estimate the stoichiometry of neutralization and entry simultaneously, rather than estimating only the stoichiometry of neutralization using independently estimated stoichiometric parameters of entry as in the previous subsection. Adopting this approach, one obtains 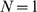 and 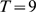 fitting the basic model to Yang's data [2]. A bootstrap routine with 1,000 replicates yielded 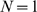 in 100% of the cases, whereas the estimate for the stoichiometry of entry was 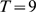 in 80%, and 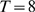 in 20% of the bootstrap replicates. Stratifying the data with respect to different antibody-binding sites, we obtain the same parameter estimates with larger confidence intervals. In particular, for antibodies binding to the CD4 binding site and the antibody 2G12 the stoichiometry of neutralization is estimated to be 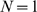 with 99.8% and 97% probability, respectively. For the other antibody classes, 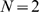 cannot be excluded at a significance level of 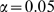.

The approach of simultaneously estimating the stoichiometry of neutralization and entry does not yield estimates with sufficient confidence for the imperfect transfection and the segregation models. Estimates with the proximity corrected model cannot be obtained due to computational constraints. The soft threshold model for entry yields parameter estimates very similar to those obtained by independent estimation of neutralization and entry parameters. Stratifying data by antibody binding site does not change this result significantly.

## Discussion

In this paper, we developed a framework for the estimation of the stoichiometry of HIV neutralization and, as an example how to apply these models, we reanalyze neutralization data [2]. As in our framework for the estimation of the stoichiometry of entry [1], we find that the distribution of trimer numbers is essential for the estimation of the stoichiometry of neutralization. A second major finding is, that the stoichiometry of neutralization may not be estimable if the variation in the number of plasmids that transfect the virus-producer cells in the generation of pseudotyped virions, or the segregation of envelope proteins within the transfected cells are too large. This is due to the fact that, in this case, virions do not express many hetero-trimers, which contain most information on the stoichiometry of neutralization. To ascertain that the experimental procedure is indeed a viable approach for the estimation of the stoichiometry of entry, the variation in transfection and segregation coefficient should be determined.

As defined in our study on HIV entry [1], the measurements for the amount of virions that productively infect a cell is the relative infectivity, RI. In contrast, Yang et al. [2] define the percent neutralization sensitivity, %NS, for studying the stoichiometry of neutralization. The relation between these variables is simply 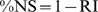. Yang's model expresses the stoichiometry of entry 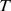 and the stoichiometry of neutralization 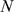 as continuous parameters. Since only an integer valued number of trimers can actually bind to the CD4-receptor and 1,2 or 3 monoclonal antibodies can bind to one trimer, 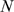 and 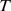 have to be discrete variables, as we modeled them in all models. The most important difference between our models and those of Yang and Klasse [2],[28] is that we include the distribution of trimer numbers. We show, that this distribution, i.e. the frequencies of virions with 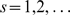 trimers, is an important input factor which affects the predictions for the relative infectivities and therefore the estimates of the stoichiometric parameters strongly.

The neutralization data of Yang et al. [2] can be analyzed in two ways. Either we use stoichiometric parameters of entry that were independently estimated to estimate the stoichiometry of neutralization from the neutralization data. Or, we attempt to estimate both, the stoichiometric parameters of entry and neutralization from the neutralization data. For the basic, proximity, and soft threshold models, we can infer the stoichiometry of neutralization. The stoichiometry of neutralization cannot be inferred from the imperfect transfection model and the segregation model if we use previous estimates for the parameters of these models. This is due to the lack of hetero-trimers according to the previously estimated parameters. Pooling over all antibodies, the fit to the data for all these models suggest that, on average, one antibody is sufficient for trimer neutralization. We also obtain a stoichiometry of neutralization of one if we stratify the data by antibody binding sites. Using the second approach in which we try to estimate the stoichiometric parameters of neutralization and entry simultaneously from the neutralization data, we find that the estimates of the stoichiometry of neutralization largely agree with those obtained with the first approach. However, the estimates for the entry parameters deviate from the parameters obtained by analyzing entry data only [1]. This is due to the extremely small differences between the predictions of the relative infectivities for some parameter combinations. Hence, we suggest to determine the entry parameters independently from experiments similar to those presented in [2].

As we suggested previously [1], a reliable estimate of the stoichiometric parameters of entry requires elucidating certain aspects of the experimental assays further. We suggest the following line of experiments (in the order of importance):

1. Determination of the variation between the fractions of mutated envelope proteins of the Env pool and mutated Env encoding plasmids and the degree of segregation. This point is even more important than the determination of the distribution of trimer numbers because the viability of experimental approach for the estimation of the stoichiometry of neutralization hinges upon low variation and segregation coefficients.
2. Determination of the trimer number distribution 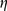. This could be done by cryoelectron tomography as in [16], [18]. As for the estimation of the stoichiometry of entry, the trimer number distribution is a very important input parameter, because it enters in all models.
3. In a last line of experiments one has to check if trimers can move freely on the virion's surface, because recent cryoelectron micoscropy tomograhical studies conclude either fixed positions of trimers on the surface [18] or free movement of trimers [16],[17]. If trimers can change their positions, the proximity model would not be valid.

These experiments allow a deeper insight into the biological processes during transfection and infection of target cells. Some models could be falsified or combined to one model, which explains the infectivity assays best. The entry parameters for this final model should be studied first and then, the stoichiometry of trimer neutralization can be studied.

In the current analysis, experimental data from 4 different virus strains neutralized by monoclonal antibodies with different specificities and mode of action are included. Due to the relatively low number of data points available, stoichiometries of individual antibodies could not be assessed. While the majority of interactions appear to follow a 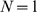 stoichiometry, we can currently not rule out that stoichiometric differences between monoclonal antibodies exist.

The stoichiometry of trimer neutralization, which is the focus of this paper, should not be confused with the single- and multi-hit model parameters proposed by McLain and Dimmock [29]. Their models aimed to determine the number of antibodies required for the neutralization of an entire virion. Since it has been established that virions differ in the number of trimers on their surface [18],[30] we cannot expect to describe virion neutralization with a single parameter. More recently, stoichiometric parameters have been proposed for the quantitative description of HIV entry and neutralization [2],[11],[14],[31]. In particular, HIV neutralization is currently described by the number of trimers on the virion's surface, the stoichiometry of entry and trimer neutralization. In combination with the stoichiometry of entry, the stoichiometry of trimer neutralization can be used to estimate the mean number of antibodies that neutralize a single virion. The number of trimers per virion varies and so does the number of trimers, which have to be neutralized. As an example, let us assume that the basic model is valid and that the stoichiometry of entry is 8, i.e. 8 functional trimers are needed to infect a target cell. Imagine a virion with 10 trimers. If two of them are neutralized, this virion is still infectious. Neutralizing one more trimer renders the virion non-infectious. In total, at least 3 trimers have to be neutralized for neutralizing the virion. Assuming that one antibody is able to neutralize one trimer, i.e. 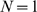, at least 3 antibodies are needed to neutralize the virion with 10 trimers. However, 3 antibodies may not be sufficient. Imagine, for example, that the 3 antibodies bind to the 3 envelope proteins of the same trimer, then only one trimer is neutralized. While we need at least 3 antibodies to neutralize this virion, 7 antibodies will be sufficient. Still assuming 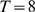 and 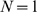, a virion with 30 trimers would be neutralized if at least 23 trimers loose their functionality. Therefore at least 23 but not more than 67 antibodies are needed. We can estimate the mean number of monoclonal antibodies required to neutralize virions with 10 trimers as 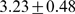 and virions with 30 trimers as 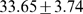 (these estimates are based on simulations with 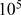 virtual virions). We plan to study how the stoichiometries of entry and neutralization relate to the neutralization of a population of viruses in the future.

The models we present here address the question of how many monoclonal antibodies are needed to neutralize a single trimer *in vitro*. *In vivo* however, there will always be a mixture of different monoclonal antibodies attacking the virions. To predict the effect of a polyclonal antibody response on virus replication, it will be necessary, in addition to estimating the stoichiometries for each antibody clone, to investigate how they synergize or antagonize each other. To illustrate what exactly we mean by synergy and antagonism assume, for example, we have two antibody clones, A and B with stoichiometries of neutralization of 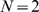, and 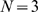, respectively. If one antibody A is bound to a trimer already, how many antibodies B are then required for neutralization? Further, does it matter where the B antibodies bind, i.e. whether they bind to the same envelope protein as antibody A or to a different one? To assess if the antibodies synergize or antagonize in this sense, one can perform experiments using pseudotyped viruses with mixed envelope proteins (very similar to those that have been conducted to estimate the stoichiometry of neutralization) in combination with our mathematical models. For this particular question, one should mix envelope proteins resistant to neutralization by antibody A with envelope proteins resistant to neutralization by antibody B. The relative infectivity of these pseudotyped viruses has to be measured under saturation of antibody A and B. If antibody A and B synergize, the relative infectivity in this experiment will be lower than if the antibodies act independently. This is because the only trimer that is not neutralized is one consisting of two envelope proteins with A-resistance and one with B-resistance. If antibodies A and B act independently these trimers are not neutralized because they bind fewer than necessary numbers of A and B. How the understanding of the stoichiometry of neutralization by a mixture of antibodies scales up to the level of the entire virion depends strongly on whether the antibody binding sites overlap. If they do, the number of antibodies required for neutralization will be lower than in the case of non-overlapping antibody binding sites because there is less opportunity for antibodies to bind uselessly.

We presented a modeling framework which enables us to investigate the number of antibodies that are needed to neutralize a single trimer and if this quantity varies between different antibodies. As the stoichiometry of trimer neutralization is the basis for the calculation of the stoichiometry of virion and population neutralization, it is an important parameter for the quantitative understanding of the protection antibodies may confer.
